# Educating primary care clinicians about health disparities

**DOI:** 10.1186/1750-4732-1-5

**Published:** 2007-02-01

**Authors:** Roberto Cardarelli, Ana L Chiapa

**Affiliations:** 1 Department of Family Medicine, University of North Texas Health Science Center, Texas College of Osteopathic Medicine, Fort Worth, Texas 76107, USA

## Abstract

Racial and ethnic health disparities inarguably exist in the United States. It is important to educate primary care clinicians regarding this topic because they have the ability to have an impact in the reduction of health disparities.

This article presents the evidence that disparities exist, how clinicians contribute to these disparities, and what primary care clinicians can do to reduce disparities in their practice. Clinicians are able to impact health disparities by receiving and providing cross-cultural education, communicating effectively with patients, and practicing evidence-based medicine. The changes suggested herein will have an impact on the current state of health of our nation.

## Background

The U.S. racial and ethnic minority population will grow from 28% in 1998 to nearly 40% in 2030 [1]. According to the Institute of Medicine (IOM), health disparities inarguably exist among racial and ethnic minorities [2]. It is important to address health disparities because consequences include poorer health, increased suffering, and higher mortality [2]. Many racial and ethnic minorities have higher mortality rates from cancer, diabetes, and cardiovascular disease [3]. African Americans have a higher cancer mortality rate (243.1 vs. 193.9 per 100,000, respectively) and twice the cardiovascular mortality rate compared to white Americans [4,5]. Among Hispanics, the diabetes death rate ranges from 47–172 per 100,000 depending on nationality (Cuban, Mexican, Puerto Rican, etc.), more than twice the rate of white Americans (23 per 100,000) [4]. Furthermore, Hispanic women have the highest cervical cancer incidence rate [6].

Health disparities have a financial toll as well. The higher burden of disease affects the health of the nation as a whole. Poorer health requires increased expenditure, especially when complications arise from uncontrolled or undetected disease. For example, African American women are more likely to have late-stage breast cancer at the time of diagnosis, more often requiring intensive treatment and hospitalization, and leading to more disability [7]. Loss of individual productivity also contributes to national health care costs, impacting all individuals regardless of race or ethnicity.

Despite concerted efforts to address and eliminate health disparities, many complicated, interrelated factors still need to be overcome. According to the IOM report, *Unequal Treatment: Confronting Racial and Ethnic Disparities in Health Care*, health disparities occur at different levels, including health care systems and their administration, clinicians and their practices, and patients themselves [2]. At the clinical level, there are several factors that may contribute to racial and ethnic health inequity [2]. Clinicians, patients, and the clinical encounter all impact health disparities. For example, a person's interaction with the clinician may lead to non-adherence, distrust, and misunderstandings that lead to poor health. Therefore, primary care clinicians have an important role and the ability to decrease health disparities [8,9].

The purpose of this paper is to expose primary care clinicians to the current state of health inequality and to describe how they may positively impact health disparities in their practice.

### How are health disparities and primary care related?

There are a variety of factors that lead to disparities in care, such as access to care, socioeconomic position, and social factors. In addition, there is evidence that clinic interactions (front desk, medical assistant, etc.) and clinician-patient encounters may lead to health disparities [2,10-12].

Primary care is the gateway to accessible health care in the United States, especially since the growth of managed care. Primary care has been defined as the provision of integrated, accessible health care services by clinicians who are accountable for addressing a large majority of health care needs, developing a sustained partnership with patients, and practicing in the context of family and community [13,14]. The importance of receiving quality care from primary care clinicians is reflected in a recent review [15]. First, health is better in areas with more primary care clinicians. Better health is characterized by lower rates of mortality, improved health outcomes, and increased lifespan. Second, people who identify a primary care clinician as their usual source of care have better health outcomes as well. Third, the characteristics of primary care are associated with better health. These characteristics are first-contact access for each need; long-term person focused care; comprehensive care; and coordinated care [15]. However, primary care access is inequitable and factors associated with the clinical encounter are related to various health inequalities which interact at different levels [12,16]. Minorities have reported poorer care compared to whites in several domains of care, such as communication, trust, accessibility to clinics, and continuity of care [17,18].

### Evidence and potential sources of health disparities

Factors that contribute to health disparities can be divided into two sets. The first set involves the operation of healthcare systems and the environment in which they operate. These factors affect access to care. Health insurance has been the most studied factor that affects access to health care. There are about 39.2 million uninsured people in the country, and minorities comprise more than 60% of that population [19]. Availability of services also affects access. Whites are the group with the highest percentage of a usual source of care, while Hispanics are the group with the lowest percentage [19].

Evidence exists of the differences in the quality of care that is received [2]. Three mechanisms by which healthcare disparities can occur at the clinical encounter are: 1) bias (or prejudice) against minorities; 2) greater clinical uncertainty when interacting with minority patients; and 3) beliefs (stereotypes) held by clinicians about the behavior or health of minorities [2].

**Healthcare provider bias **can occur unconsciously. Research has found that prejudicial attitudes still remain common in America [2], and that clinicians' diagnostic and treatment decisions may be influenced by the patients' race or ethnicity. For example, physicians were found to be less likely to recommend catheterization procedures to African American females compared to white males and females, and African American males [20]. Physicians were also found to rate African American patients as less intelligent, less educated, more likely to abuse drugs and alcohol, more likely to not follow medical advice, and less likely to participate in cardiac rehabilitation than white counterparts [21]. Although there are many factors influencing clinician decisions, subtle factors such as bias may have an effect on the patients and their health outcomes. Primary care clinicians need to become aware of unconscious and unintentional actions or decisions in order to make changes in the way they provide care.

**Clinical uncertainty **occurs when clinicians make decisions about the severity of an illness based on prior beliefs or experience [2]. These prior beliefs and experiences will be different depending on the age, gender, socioeconomic status, race and ethnicity of the patient. If the clinician does not have the information needed to make a diagnostic decision, (for example, if the clinician has difficulty understanding the symptoms), then the clinician will be more likely to use prior beliefs and experiences to make diagnostic and treatment decisions. As a consequence, the patient's needs may not be met.

**Stereotypes **can be defined as categories that people use (sex, race, etc.) to process and recall information about others [22]. People then use the information in these categories to understand and simplify complex situations. Although explicit stereotyping is rarely seen these days, it still exists in more implicit and subtle ways. Even people who do not believe they are prejudiced often demonstrate implicit or unconscious bias or stereotypes.

Clinicians must become aware that they are not exempt from unintentional (or intentional) bias or discrimination when caring for patients. Most clinicians strongly refute the idea that they provide differential care to ethnic and racial minorities [2]. However, it is usually small recurrent unintentional acts during the clinician-patient encounter that may contribute to existing health disparities [2]. Awareness by the clinic staff and clinicians is one of many concerted efforts that are needed to reduce health disparities in this country.

Quality medical care is often influenced by system factors outside of the clinician's control, such as time restrictions, cost-containment pressures, insurance status and ability to pay. However, it is important for primary care clinicians to be vigilant and address these issues in order to provide equal and comprehensive medical care regardless of an individual's age, race, ethnicity, gender, and socioeconomic position [2].

### What can primary care clinicians do to address health disparities?

There are several things that primary care clinicians can do in their practice to aid in national efforts to reduce health disparities. Clinicians can receive and provide cultural competence/cross-cultural education, learn how to communicate effectively with patients, and practice evidence-based medicine.

#### Cross-cultural education

Education about different cultures can be used to avoid stereotypes, bias, and clinical uncertainty. Students and clinicians may greatly benefit from cross-cultural education or training. However, clinicians should be aware that achieving cultural competence is a process, and does not happen from one day to another with a textbook, or as a quick fix. Cross et al (Table 1) developed a framework in which cultural competence occurs in a continuum and in six stages: 1) Cultural Destructiveness, 2) Cultural Incapacity, 3) Cultural Blindness, 4) Cultural Pre-competence, 5) Cultural Competency and 6) Cultural Proficiency [23]. An awareness of one's own position within the different stages is the first step to achieving full cultural competence.

**Table 1:** Stages of Cultural Competence

**Cultural Destructiveness**	Characterized by attitudes, policies, structures, and practices that are destructive to other cultures. They are dehumanizing of other people, and assumptions of superiority are prevalent. This stage occurs consciously.
**Cultural Incapacity**	This stage occurs when there is unintentional cultural destructiveness, bias, paternalism, ignorance, and/or fear.
**Cultural Blindness**	Involves a philosophy of being unbiased, treating all people the same, belief that culture, class or color does not make a difference. People in this stage are well-intentioned; however, it is still ethnocentric.
**Cultural Pre-competence**	Characterized by the realization of weaknesses and gaps that are missing when working with other cultures. There is a desire for inclusion, a commitment to civil rights, and a desire to implement training. However, there may be a danger of false accomplishment.
**Cultural Competency**	Characterized by an acceptance and respect for differences. There is a continual inquiry about other cultures and an expansion of knowledge.
**Cultural Proficiency**	Last stage where all cultures are held in high esteem and there is a responsibility taken for constant development of new knowledge and approaches to interaction. This stage assumes responsibility to transfer skills and advocate cultural competence to others within a system or an organization.

Adapted from Cross et al [25]

The Office of Minority Health published the Culturally and Linguistically Appropriate Services Standards (CLAS) in 2000 [24]. One of the main themes of the standards is culturally competent care. Cultural competence is defined as a set of congruent behaviors, attitudes, and policies that come together in a system, agency, or among professionals that enables effective work in cross-cultural situations [24]. Culture refers to the patterns of behavior in humans that include language, thoughts, communication, actions, customs, beliefs, values, and institutions of race, ethnicity, religion, or social groups [25]. Culture not only refers to race, ethnicity, and religion, but also refers to gender, sexual orientation, age, disability, and socioeconomic status [24]. Educational programs should have a patient-centered focus, where the patient is the center of attention, rather than the patient's cultural group characteristics, or the disease itself.

However, many training programs use a categorical approach to teaching cultural competence by focusing on certain groups of people. Carrillo, Green, and Betancourt recommend an emphasis on the differences between individual patients, rather than groups, in cross-cultural curricula [26]. There are many different models of cultural competency/cross-cultural curricula that are currently being used in medical schools. Examples of such curricula and suggested readings in the topic are presented in Table 2[26-29].

**Table 2:** Cross-Cultural Resources

**Cross-Cultural Trainings**
Carrillo JE, Green AR, Bethancourt JR: **Cross-cultural primary care: A patient-based approach. ***Annals of Internal Medicine *1999, **130: **829–834.
Culhane-Pera KA, Reif C, Egli E, Baker NJ, Kassekert R: **A curriculum for multicultural education in family medicine. ***Family Medicine *2006, **29: **719–723.
Kristal L, Pennock PW, Foote SM, Trygstad CW: **Cross-cultural family medicine residency training. ***Journal of Family Practice *1983, **17: **683–687.
Clark L, Thornam C: *Healthcare in multicultural environments*. Boulder, CO: University of Colorado School of Nursing; 1998.
**Cross-Cultural Readings**
Galanti G: *Caring for patients from different cultures*. 3rd ed. Philadelphia: University of Pennsylvania Press; 2004.
Purnell LD, Paulanka BJ: *Transcultural health care: a culturally competent approach*, 2nd Edition edn. Philadelphia: F.A. Davis; 2003.
Gropper RC: *Culture and the clinical encounter: An intercultural sensitizer for the health profession*. Yarmouth, ME: Intercultural Press; 1996.
Rundle A, Carvalho M, Robinson M: *Cultural Competence in Health Care: A practical Guide*. San Francisco CA: Jossey-Bass; 1999.
Spector R: *Cultural Diversity in Health and Illness*, 6th ed. Prentice Hall; 2003.

#### Communication

Many racial and ethnic minorities, especially limited English-speaking minorities, report poor communication with their clinicians and have more problems with different aspects of the clinician-patient relationship [30-32]. Many patients who experience poor communication are less likely to follow instructions, take medications, and follow-up with tests and appointments, all leading to poorer health [33-35]. Thus, effective communication is another strategy primary care clinicians can use to reduce health disparities. Effective communication can be defined as using little medical jargon, speaking clearly, and ensuring the patient understands the given information [36]. Stereotypes can be avoided if the clinician is able to gather accurate information about whether the patient understands his or her condition. Kleinman and colleagues developed a set of interviewing questions to elicit how patients understand their condition [37]. These patient-centered questions are presented in Table 3 and can help clinicians understand and address the patient's ailments.

**Table 3:** Patient-Centered Interview Questions

What do you call the illness?
What do you think has caused the illness?
Why do you think the illness started when it did?
What problems do you think the illness causes? How does it work?
How severe is the illness? Will it have a long or short course?
What kind of treatment do you think is necessary?
What are the most important results you hope to receive from this treatment?
What are the main problems the illness has caused you?
What do you fear most about the illness?
Adapted from Kleinman et al [37]

Complex language can have a negative effect on successful communication between a clinician and patient. A report by the IOM found that the complex language that clinicians use to communicate with patients, either verbally or written, is a problem for many patients, not just recent immigrants or those with a low level of education [38]. Termed "health literacy," this important concept must be taken into account when communicating with patients.

Health literacy is defined by the National Library of Medicine and Healthy People 2010 as the "degree to which individuals have the capacity to obtain, process, and understand basic health information and services needed to make appropriate health decisions" [38-41]. Many factors affect health literacy, such as the patient's level of education, cultural background, and native language. The clinician's ability to effectively and appropriately communicate with the intended audience is also important [38]. Even people with strong health literacy skills have difficulty understanding written information from clinicians, such as patient information sheets and prescription drug labeling [38]. If patients have difficulty understanding instructions given by a clinician, they may not be able to understand their health condition, may have difficulty with treatment decision making, and may not take their medications correctly [38]. A patient centered approach, as shown in Table 3, where the patient's perspectives, values, beliefs, and behaviors are taken into account may reduce these communication barriers.

Clinicians and patients who do not speak the same language substantially complicate communication issues. Using trained interpreters is the best way to ensure that patients understand information that is given to them. If non-trained interpreters are used, such as family members or employees who are pulled from their regular job to interpret who are not aware of the potential problems that may arise, problems of lost information, misunderstandings, and miscommunication may occur. This may result in patients not having their needs addressed, requiring returned clinic visits, ordering unnecessary tests, or even misinterpretations regarding prescribed drugs. The Cross Cultural Health Care Program (CCHCP) developed guidelines to help clinicians work through an interpreter [42]. These guidelines state that the decision to use an interpreter is made whenever the clinician feels that language or cultural differences may cause a barrier to clear communication, or whenever a patient requests an interpreter. Choosing an interpreter may also be a challenge. The CCHCP makes several suggestions as to how to choose an interpreter. First, make sure that the interpreter is fluent in both languages; testing may be needed. Second, make sure the interpreter is trained as an interpreter. The fact that a person is bilingual does not make her or him an interpreter; there are special skills involved. Third, do not use a family member. Family members often edit the patient's message, add their own opinions, and answer for the patient. Fourth, never use a child. This creates role reversal and power reversal, and it should not be the responsibility of a child to relay bad news to parents or family members.

The CCHCP also provides suggestions on how to work through an interpreter [43]. First, request interpretation of everything, and in the first person. Second, speak directly to the patient, not to the interpreter. Third, insist that everything you say is interpreted, as well as everything that the patient says, or that family members say. Fourth, be patient. Providing care through an interpreter often takes longer. However, this will avoid wasted time, misunderstandings, or unnecessary tests.

Some organizations or clinicians' offices may be too small to hire a full time interpreter or there may be barriers to hiring bilingual staff. In such cases, another option would be using the American Telephone and Telegraph (AT&T) language line [44]. The service may be used by a subscribed client or company, or may be used by an unsubscribed individual for less frequent use. Although at first glance the price for this service may seem quite expensive (ranging from $2.20 per minute to $7.25 per minute), it becomes cost-efficient in the long run because clinicians will have a better understanding of the patients' symptoms, conditions, and life styles. Patients will also have a better understanding of their condition and their medications, and will be less likely to return due to misunderstandings.

#### Practicing Evidence-Based Medicine

The use of evidence-based medicine (EBM) can be another method to reduce health disparities. According to the University of Toronto Center for Evidence-Based Medicine, EBM is the integration of best research evidence that is clinically relevant with clinical expertise and patient values [45]. The need for valid information, the inadequacy of current resources, and the lack of time to spend with the patient are some reasons why interest in EBM has increased in the past years [46]. EBM can also reduce clinician bias and stereotypes by ensuring that practice is based on one's expertise and the most current applicable evidence. Adherence to evidence-based guidelines allows clinicians to make decisions that are reflective of current research findings, avoiding conscious or unconscious decisions based on bias or stereotypes. However, there are many realities that must be considered. When serving low income patients and/or individuals from underserved populations, resources may be severely limited. Utilizing the best evidence that fits the clinician's practice environment and special circumstances is recommended. For example, clinicians practicing in non-profit free clinics must make strategic and economic decisions when deciding what medication to prescribe because medications are often out-of-pocket expenses for patients. Nonetheless, studies have shown that practicing EBM has economic advantages as well. The lack of compliance with antihypertensive guidelines, by using second-line medications over first-line medications (such as hydrochlorothiazide), was associated with potential increases in health care expenditures in the range of $2.6 billion to $3.2 billion in 1996 [47]. Numerous EBM resources are available on the Internet to allow primary care clinicians to keep abreast of EBM guidelines (Table 4). Use of EBM principles may potentially increase health equity among patients.

**Table 4:** Useful Evidence-based Medicine Web Sites

Agency for Healthcare Research and Quality Guideline Resources	
American College of Physicians Journal Club	
Bandolier Journal	
Cochrane Collaboration	
Database of Abstracts of Reviews of Effects (DARE)	
Evidence-based Medicine Journal	
Evidence Syntheses and Systematic Evidence Reviews (USPSTF)	
Guide to Clinical Preventive Services, 2005 (USPSTF)	
National Guideline Clearinghouse	
PubMed	

Although the recommendations provided may not be simple to implement, primary care clinicians can have a significant role in reducing health disparities through incremental changes. Education is the key to understanding patients' perspectives and providing a higher quality of care. Other steps that can be taken are conscious efforts to communicate with patients more clearly and using trained interpreters when needed. Also, communication style, such as asking questions in a more caring manner or validating a patient's concern, may have a positive impact on the health of patients. The use of EBM may be beneficial, not only for the populations that experience health disparities, but also for the patient population as a whole, reducing costs and increasing equity. The sum of our small changes, taken together, will make a significant impact.

## Abbreviations

AT&T: American Telephone and Telegraph

CCHCP: Cross Cultural Health Care Program

CLAS: Culturally and Linguistically Appropriate Services Standards

DARE: Database of Abstracts of Reviews of Effects

EBM: Evidence-Based Medicine

IOM: Institute of Medicine

U.S.: United States

## Competing interests

The author(s) declare that they have no competing interests.

## Authors' contributions

RC is the director for the Division of Research, Center for Evidence-Based Medicine, and the North Texas Primary Care Practice-Based Research Network. RC drafted the manuscript and approved the final version.

ALC is the research associate for the Division of Research, and is the research coordinator for the North Texas Primary Care Practice-Based Research Network. AC drafted the manuscript and approved the final version.
